# Supplementary material for: Limited-View and Sparse Photoacoustic Tomography for Neuroimaging with Deep Learning
Source: Sci Rep. 2020 May 22;10:8510. doi: 10.1038/s41598-020-65235-2 (PMC7244747; doi:10.1038/s41598-020-65235-2)
Supplement: Supplementary file 1 — Supplementary Figures. [file 41598_2020_65235_MOESM1_ESM.pdf]

Limited-View and Sparse Photoacoustic Tomography for Neuroimaging with Deep Learning

Steven Guan, Amir A. Khan, Siddhartha Sikdar, and Parag V. Chitnis

Supplementary Figures

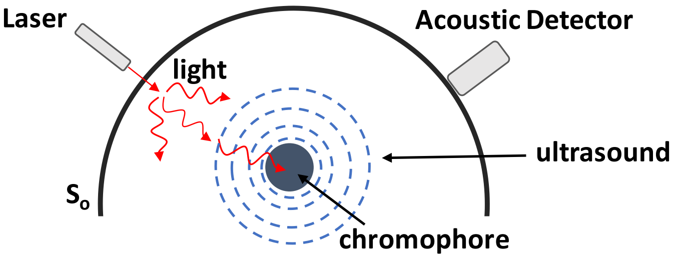

Supplementary Fig. 1 | Diagram of Limited-View and Sparse PAT. The biological tissue is illuminated with a pulsed nanosecond laser. Chromophores within the tissue absorbs the scattering light and undergo thermoelastic expansion resulting in the generation of ultrasound. Acoustic sensors along the measurement surface S_o that partially encloses the chromophores are used to detect the emitted ultrasound.

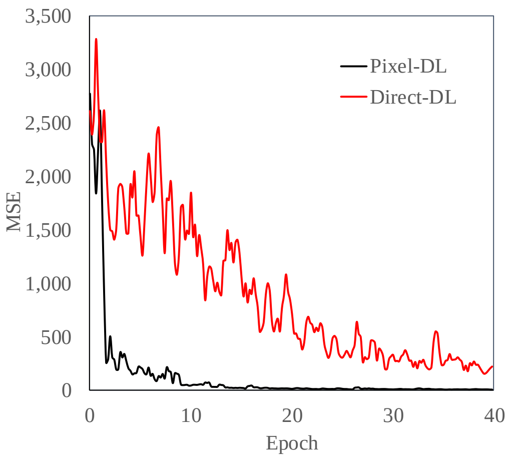

Supplementary Fig. 2 | Mean Squared Error (MSE) Training loss for Pixel-DL and mDirect-DL. Pixel-DL converged faster and to a lower MSE loss compared mDirect-DL. The mDirect-DL CNN did learn to reconstruct an image from the interpolated sensor data but learned features that did not generalize and overfitted to the training data.

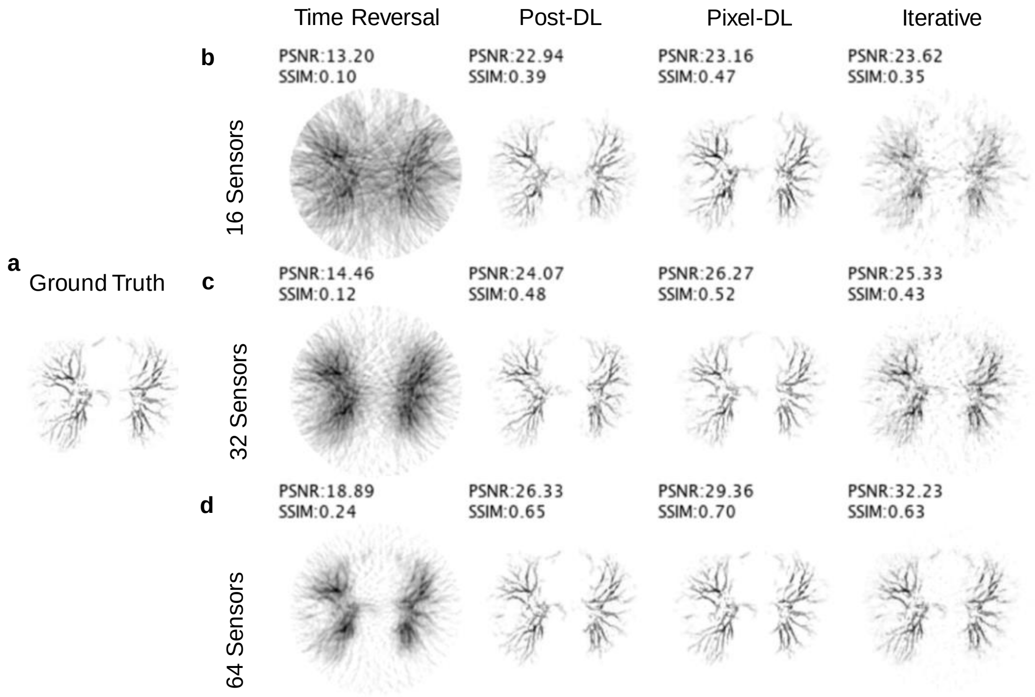

Supplementary Fig. 3 | Limited-view and sparse PAT image reconstruction of lung vasculature. PAT sensor data acquired with a semi-circle limited-view sensor array at varying sparsity levels. **a,** Ground truth image used to simulate PAT sensor data. **b,** PAT reconstructions with 16 sensors. **c,** PAT reconstructions with 32 sensors. **d,** PAT reconstructions with 64 sensors.

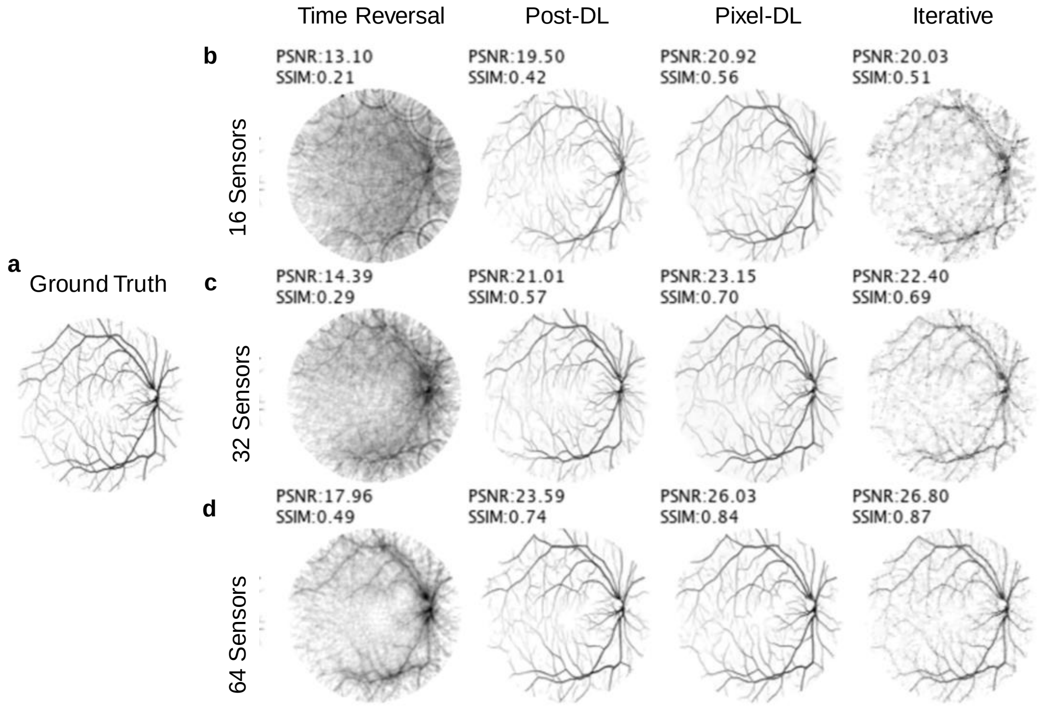

Supplementary Fig. 4 | Limited-view and sparse PAT image reconstruction of fundus vasculature. PAT sensor data acquired with a semi-circle limited-view sensor array at varying sparsity levels. **a,** Ground truth image used to simulate PAT sensor data. **b,** PAT reconstructions with 16 sensors. **c,** PAT reconstructions with 32 sensors. **d,** PAT reconstructions with 64 sensors.
